# Impact of nutritional interventions among lactating mothers on the growth of their infants in the first 6 months of life: a randomized controlled trial in Delhi, India

**DOI:** 10.1093/ajcn/nqaa383

**Published:** 2021-02-10

**Authors:** Sunita Taneja, Ravi Prakash Upadhyay, Ranadip Chowdhury, Anura V Kurpad, Himani Bhardwaj, Tivendra Kumar, Pratibha Dwarkanath, Beena Bose, Sarita Devi, Gunjan Kumar, Baljeet Kaur, Rajiv Bahl, Nita Bhandari

**Affiliations:** Center for Health Research and Development, Society for Applied Studies, New Delhi, India; Center for Health Research and Development, Society for Applied Studies, New Delhi, India; Center for Health Research and Development, Society for Applied Studies, New Delhi, India; Department of Physiology, St John's Medical College, Bengaluru, India; Center for Health Research and Development, Society for Applied Studies, New Delhi, India; Center for Health Research and Development, Society for Applied Studies, New Delhi, India; Department of Physiology, St John's Medical College, Bengaluru, India; Department of Physiology, St John's Medical College, Bengaluru, India; Department of Physiology, St John's Medical College, Bengaluru, India; Center for Health Research and Development, Society for Applied Studies, New Delhi, India; Center for Health Research and Development, Society for Applied Studies, New Delhi, India; Department of Maternal, Newborn, Child, and Adolescent Health, World Health Organization, Geneva, Switzerland; Center for Health Research and Development, Society for Applied Studies, New Delhi, India

**Keywords:** maternal nutrition, micronutrients, lactation, infant growth, maternal health, randomized controlled trial, India

## Abstract

**Background:**

In lower-middle-income settings, growth faltering in the first 6 mo of life occurs despite exclusive breastfeeding.

**Objective:**

The aim was to test the efficacy of an approach to improve the dietary adequacy of mothers during lactation and thus improve the growth of their infants.

**Methods:**

Eligible mother–infant dyads (infants ≤7 d of age) were randomly assigned to either intervention or control groups. Mothers in the intervention group received snacks that were to be consumed daily, which provided 600 kcal of energy—with 25–30% of energy derived from fats (150–180 kcal) and 13% of energy from protein (80 kcal). Micronutrients were supplemented as daily tablets. We provided counseling on breastfeeding and infant-care practices to mothers in both groups. The primary outcome was attained infant length-for-age *z* scores (LAZ) at 6 mo of age. Secondary outcomes included exclusive breastfeeding proportion reported by the mother, maternal BMI and midupper arm circumference (MUAC), hemoglobin concentrations in mothers and infants, and the proportion of anemic infants at 6 mo of age.

**Results:**

We enrolled 816 mother–infant dyads. The intervention did not achieve a significant effect on LAZ at 6 mo (adjusted mean difference: 0.09; 95% CI: −0.03, 0.20). Exclusive breastfeeding at 5 mo was higher (45.1% vs. 34.5%; RR: 1.31; 95% CI: 1.04, 1.64) in the intervention group compared with the controls. There were no significant effects on mean hemoglobin concentration or the proportion of anemic infants at 6 mo of age compared with the control group. We noted significant effects on maternal nutritional status (BMI, MUAC, hemoglobin concentration, and proportion anemic).

**Conclusions:**

Postnatal supplementation of 600 kcal energy, 20 g protein, and multiple micronutrients daily to lactating mothers did not affect infant LAZ at age 6 mo. Such supplementation may improve maternal nutritional status. This trial was registered at Clinical Trials Registry–India as CTRI/2018/04/013095.

## Introduction

The first 6 mo of life epitomize a transition from the neonatal period to childhood—during which growth and neurologic and immunologic development occur rapidly (1). It is assumed that adequate nutrition is ensured during the first 6 mo of life by breastfeeding; however, recent evidence suggests that undernutrition occurs before this time and is associated with increased risk of mortality and growth failure in later life (2–4). High rates of undernutrition (20–30%) among 0- to 6-mo-old infants were also reported in the Indian National Family Health Survey-4 (2, 5).

Exclusive breastfeeding in the first 6 mo of life is beneficial for survival, a reduction in infections, and optimal neural development (6–8). However, a recent systematic review and meta-analysis of 35 published studies showed no significant beneficial effects of promoting breastfeeding on weight or length attained by children (9). A substantial proportion of infants experience growth faltering during the first 6 mo of life in India and other Southeast Asian countries (10). This faltering occurs even in infants who do not manifest low weight and length at birth, which indicates that environmental factors contribute to early growth failure—making a compelling case for considering interventions beyond the promotion of exclusive breastfeeding.

**FIGURE 1:**
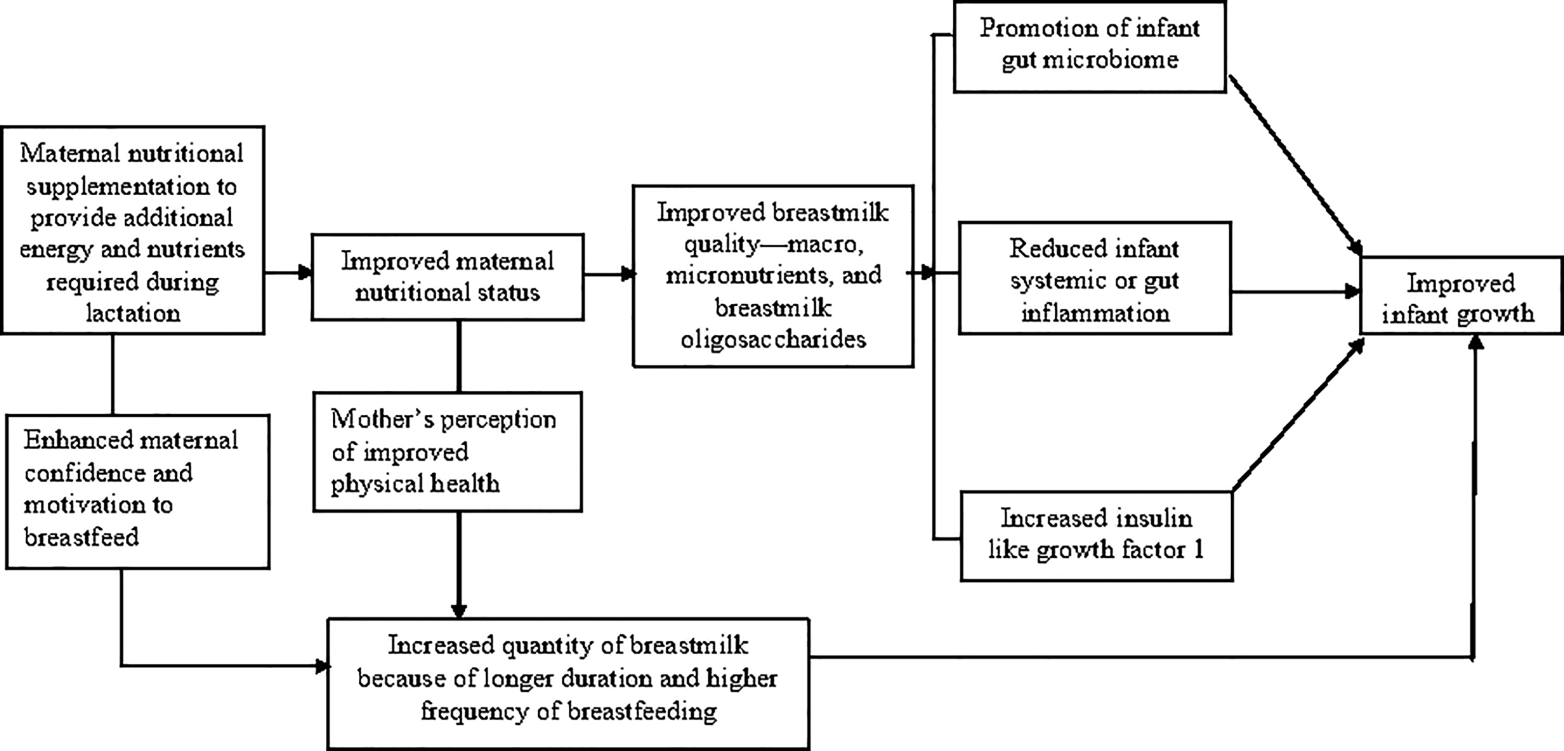
Conceptual model illustrating pathways by which supplementation during lactation might influence infant growth.

The contribution of maternal nutritional status and dietary intake to growth faltering may be far more important than is currently assumed. It is important to note that pre-existing deficiencies are common, as ∼20% of Indian mothers have a low BMI and ∼50% exhibit anemia and other micronutrient deficiencies (5, 11). Lactation increases nutritional demands, and this may further exacerbate the gaps in the nutrient adequacy of Indian mothers (12). Given the overwhelming advantages of exclusive breastfeeding, a strategic option to increase nutrient intake by infants during their first 6 mo of life would be to improve nutrient intake of the mothers. Studies have shown that, while breast-milk output is largely unaffected by maternal factors, macronutrient and micronutrient composition of breast milk beyond 3 mo of age might be affected by maternal nutritional status and dietary intake (13, 14). More specifically, while protein, lactose, and fat concentrations in breast milk remain relatively stable, maternal supplementation may influence fatty acid composition, especially of the PUFAs (e.g., DHA), as well as concentrations of thiamin; vitamin B-12; riboflavin; vitamins B-6, A, D, E, and K; iodine; and selenium (15). The fact that this possibility of altering breast-milk composition through maternal nutritional supplementation coincides with the timing of infant growth faltering raises an interesting point that high-quality nutritional supplements for the mothers may help in improving linear growth in infants. In addition to potentially improving breast-milk quality, supplementation may also improve maternal perception of physical health, and enhance a woman's confidence and motivation to breastfeed. In **Figure 1**, we present a conceptual framework that illustrates the pathways through which maternal supplementation during lactation might influence infant growth outcomes.

The present study is primarily aimed at testing the efficacy of an approach designed to improve dietary adequacy by supplementing additional energy, protein, and micronutrients during lactation. Through the administration of high-quality nutritional supplements to lactating mothers in the first 6 mo postpartum, we hoped to improve the growth of the infants at 6 mo of life. Additionally, the effects on maternal BMI, hemoglobin concentration, and the proportion with anemia were assessed in mothers and infants after the 6-mo supplementation period.

## Methods

### Study setting, design, and participants

We conducted an individually randomized, controlled efficacy trial in low-resource settings of urban Delhi, India. Study subjects were mother–infant dyads, with infants initiated into breastfeeding and enrolled within 7 d of birth. Infants whose mothers had died or did not remain with the infant, those not initiated into breastfeeding by 7 d of age, those whose mothers exhibited chronic illness requiring prolonged medical treatment, infants requiring prolonged medical attention, those with major congenital malformations, and mother–infant dyads who were likely to move out of the study site within 6 mo were excluded from the study.

### Screening and enrollment

A door-to-door survey was conducted to identify pregnant women and infants ≤7 d of age. The women identified were followed up until delivery, with more frequent contacts in the third trimester. For facility births, the screening and enrollment team visited the family once the infant arrived at home; while for those who delivered at home, the team visited as soon as possible after birth. During screening, the team member explained the study to the mother and family members and—for those who were willing—consent for screening was obtained from the mother. The mother and infant were assessed for eligibility and, if eligible, the screening and enrollment team requested group assignment through a Web-based system.

### Randomization, allocation, and blinding

Eligible mother–infant dyads were randomly assigned to either the intervention or control group through a randomization list using blocks of variable length. The list was prepared offsite by a statistician based at the WHO, Geneva, Switzerland, who was not otherwise involved with the study. The allocation was via a Web-based system. A baseline form containing socioeconomic characteristics of the family was filled out for enrolled participants. The team obtained anthropometric measurements for infants [length, weight, midupper arm circumference (MUAC), and head circumference] and their mothers (height and weight). Only 1 mother–infant dyad was enrolled from a household. Although blinding of the study teams was not possible, the supplement delivery and counseling teams had limited interaction with the independent outcome-ascertainment team.

### Study interventions

Mothers in the intervention group were provided a food supplement in the form of a snack to be consumed daily that provided 600 kcal with 25–30% of energy (150–180 kcal) from fats and 13% of energy from proteins (80 kcal). The snack contained 20 g of protein from a mix of plant- and animal-source proteins, with ∼30% (5.4–6 g) of the protein coming from a dairy source. Micronutrient supplementation was provided for a period of 180 d as daily tablets that provided 80–100% of the RDA of vitamins A, D, E, C, B-6, B-12, and C; thiamin; riboflavin; niacin; folate; iron; zinc; iodine; selenium; and copper (16).

The food supplements were prepared by Hungry Foal (https://www.hungryfoal.com/), a for-profit organization located in Gurugram, Haryana, India, and provided in the form of locally acceptable snacks. The names and ingredients of the snacks are depicted in **Table 1**. The snacks were pretested for acceptability among women in the study population before study initiation, and ultimately 5 sweet and savory snacks were selected for use in the study. Mothers were given the snack of their choice, with an option to change their preference at the time of weekly replenishment. To minimize intrahousehold sharing, snacks were promoted to be used exclusively by lactating mothers, with labels portraying a lactating woman. Timing of the mother's meals was assessed, and the optimal time for the consumption of snacks was negotiated at enrollment such that they did not replace her regular meals. The composition of multiple micronutrient (MMN) tablets was similar to the UNICEF/WHO/United Nations University international MMN preparation (UNIMMAP), and tablets were donated by The Vitamin Angel Alliance, Inc. (Vitamin Angels), California (https://www.vitaminangels.org/) (17). The composition of the MMN tablets is shown in **Supplemental Table 1**.

**TABLE 1:** Ingredients in the culturally acceptable snacks used in the present study^1^

Snack name	Ingredients
Choco Energy Bites (Hungry Foal)	Oats, sugar, malt, vegetable oil, milk solids, liquid glucose, peanuts, almond, cocoa powder
*Panjeeri* (Hungry Foal)^2^	Wheat flour, sugar, vegetable oil, milk solids, fox nuts, almond, peanuts
*Jeera* crackers (Hungry Foal)^3^	Wheat flour, water, oats, vegetable oil, milk solids, sugar, cumin, salt
Nut mixture	Oats, peanuts, rice flakes, honey, raisins, soyabean oil, milk solids, almonds, salt, spices
Biscuit	All-purpose wheat flour, sugar, edible vegetable fat, starch, soya protein isolate, milk solids, inverted sugar syrup, cream powder, cocoa powder

1 The shelf life of each of the snacks was 90 d.

2 *Panjeeri*: traditional snack considered as a nutritional supplement for lactating mothers.

3 *Jeera* crackers: name derived from the ingredient “cumin” (*Jeera* in the local language) because of its predominant taste in the snack.

During monthly visits, the counseling team visited the control- and intervention-group mothers and provided counseling on the importance of exclusive breastfeeding, infant-care practices that included early care-seeking for illness, maternal postpartum care, and optimal nutrition for the mother. This team also provided lactation-education support to mothers to promote exclusive breastfeeding. Mothers in both groups were counseled on the availability of iron–folic acid, calcium, and vitamin D through the national program (12, 18). Additionally, mothers in the intervention group were counseled monthly by nutritionists on the importance of supplement intake; and suboptimal intakes were discussed to identify any barriers to appropriate intake. The supplement-delivery team visited households to provide snack packets for 1 wk and MMN tablets for 1 mo to mothers in the intervention group. During the weekly visits, the empty snack packets were also collected. Data on compliance with respect to MMN tablets were collected each month through a tablet count.

### Sample size

Assuming an SD of 0.20 (for a 0.53-cm length, 1 SD = 2.67 cm) (19) for mean differences in attained length-for-age *z* scores (LAZ) at 6 mo of infant age between the intervention and control groups, 80% power, a 1-sided 5% ɑ level, and 10% loss to follow-up, we required a total of 340 infants per group—i.e., a total of 680 mother–infant pairs. Based on the observation of a higher-than-assumed (∼15–20%) loss to follow-up due to outmigration, the investigators approached the Technical Advisory Group (TAG). The TAG then recommended increasing the sample size by 20% to ensure adequate statistical power, and the sample size was revised to 816—i.e., 408 each in the control and intervention groups.

### Outcomes and their ascertainment

The primary outcome was attained LAZ at 6 mo of age. The secondary outcomes were attained infant weight-for-age *z* score (WAZ), weight-for-length *z* score (WLZ), MUAC *z* score (MUAC-Z), and head circumference *z* score at 6 mo of age; the proportion of infants showing stunting (LAZ < −2), wasting (WLZ < −2), and underweight (WAZ −2) at 6 mo of age; and the changes in LAZ, WLZ, and MUAC-Z at 0–3 and 4–6 mo of age.

Additional secondary outcomes were maternally reported exclusive breastfeeding proportion at 1, 3, and 5 mo of infant age; maternal BMI and MUAC at 6 mo of infant age; and hemoglobin concentration in mothers and infants and the proportion with anemia at 6 mo of infant age. Dietary intake by both the intervention- and control-group mothers was assessed using 24-h dietary recall at 3 mo of infant age.

Outcomes were assessed by an independent outcome-ascertainment team that was kept unaware of group allocation in order to reduce measurement bias. The team was trained and standardized in anthropometric measurements, and inter- and intraobserver standardization exercises were conducted at the beginning of the study and at 3-mo intervals thereafter. The team visited the households in pairs and performed anthropometric assessments at monthly intervals, from enrollment until 6 mo of infant age. Weights and lengths were taken by a pair of workers using digital weighing scales (model 354; Seca) and infantometers (model 417; Seca) to the nearest 10 g and 0.1 cm, respectively. Head circumference and MUAC were quantified using measuring tapes (model 212; Seca). The team also measured maternal height and weight at 6 mo of infant age using Seca-213 stadiometers to the nearest 0.1 cm and Salter 9509 weighing scales to the nearest 0.1 kg. Data on breastfeeding were determined by nutritionists through a 24-h recall using a structured questionnaire. Blood samples at the end of the 6-mo intervention period were collected from the mothers and their infants at home by trained phlebotomists. The anemia assessment was performed using capillary blood with a HemoCue Hb 201 Plus analyzer (20). All of the study staff received training in Good Clinical Practice guidelines.

### Ethics approval and consent to participate

This study was approved by the Ethics Committee of the Centre for Health Research and Development, Society for Applied Studies, India. Written informed consent was obtained in the local language from the caregivers before enrollment, and the study was registered in Clinical Trials Registry–India (CTRI/2018/04/013095).

### Statistical analysis

We performed all of the analyses using STATA, version 16.0 (StataCorp). The mean ± SD or median (IQR) was calculated for continuous variables and proportions calculated for categorical variables. Comparisons of means, medians, and proportions by groups were used to assess whether the randomization scheme resulted in comparability between groups. For each participant, compliance with regard to snacks or MMN tablets was presented as a percentage (%) and was calculated as total packets or tablets of MMNs consumed divided by supplement packets or MMN tablets that should have been consumed, and multiplying the resulting fraction by 100. Mothers with hemoglobin <12 g/dL and infants with hemoglobin <11 g/dL were considered to have anemia (21). Exclusive breastfeeding was defined as the infants having access to no other food or drink (including water), except for breast milk (including expressed milk), but with allowances for the infants to receive medicines, vitamins, and minerals (22).

Our primary analysis consisted of the comparison of outcomes between study groups and was based on the intention-to-treat principle. For binary outcomes, we used a generalized linear model (GLM) of the binomial family with a log-link function to calculate the effect size (RR ratio and 95% CI). For continuous outcomes, GLMs of the Gaussian family with an identity-link function were used to calculate the effect size (difference in means and 95% CIs). Purposive selection of variables for adjustments to the model was performed—that is, those variables that brought at least a 15% change in the univariate effect size between exposure (study groups) and outcome were considered for adjustment (23, 24). We included covariates in the model to narrow the CIs for the effect estimates. Although not prespecified, we evaluated effects of the intervention in subgroups in which supplementation might exert larger effects on infant growth. An exploratory analysis relating to the primary outcome (i.e., attained LAZ at 6 mo of infant age) was conducted with maternal height (<150 cm or ≥150 cm) and BMI categories (in kg/m^2^; <18.5, 18.5–24.9, or ≥25.0) and subgroups based on infant weight at enrollment (<2500 g or ≥2500 g) and stunting (LAZ < −2), wasting (WLZ < −2), and underweight (WAZ < −2) status at birth.

We also built generalized estimating equation (GEE) models for changes in anthropometric indices from enrollment to 3 mo and from 3 to 6 mo. This approach accounted for interdependence between multiple measurements in the same infant. We used GEE models of the Gaussian family with an identity-link function, an exchangeable correlation structure with intervention-by-time as interaction term. We also estimated mean 3-monthly changes of anthropometric indices as intervention-by-time interaction terms were not significant (*P* < 0.05). For all of the models, we specified a robust estimator of variance and an exchangeable correlation structure. In addition, for all of the analyses, effect sizes were reported with a 95% CI and a 2-sided *P* value <0.05 was considered to show statistical significance.

## Results

**FIGURE 2:**
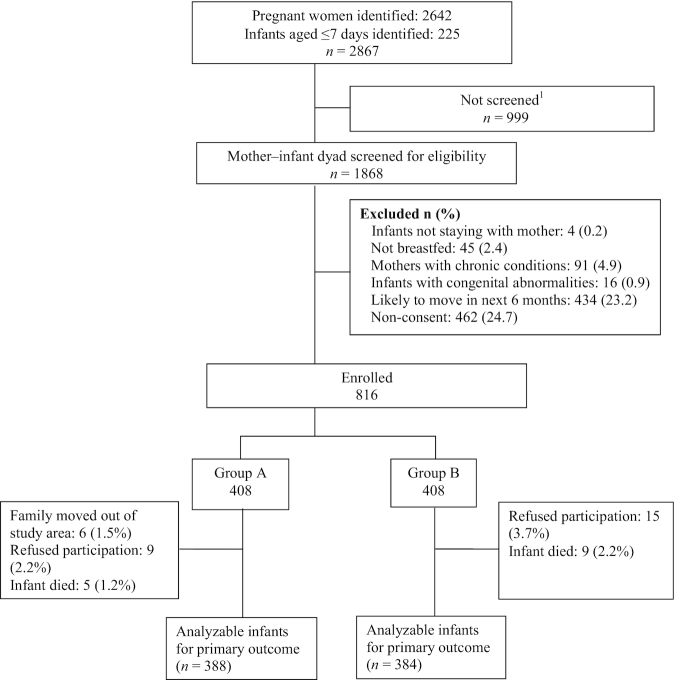
Trial profile. ^1^Delivered outside Delhi (*n* = 487), infant died (*n* = 74), stillbirth (*n* = 16), mother and infant hospitalized during first 7 d of age (*n* = 140), not visited for screening as the required 816 mother–infant dyads were enrolled (*n* = 19), family not available (*n* = 226), refused participation (*n* = 37).

Between 9 May 2018 and 31 May 2019, a total of 2642 pregnant mothers and 225 infants <7 d of age were identified through a household survey. A total of 1868 mother–infant dyads were screened for eligibility (1705 from the 2642 pregnant mothers and 163 from the 225 infants aged <7 d who were identified). We excluded 1052 mother–infant dyads because they did not meet our eligibility criteria or the family did not provide consent to participate, and thus 816 were ultimately enrolled. These mother–infant dyads were randomly assigned to either the intervention (*n* = 408) or control (*n* = 408) group (**Figure 2**). **Table 2** shows the baseline characteristics of the infants, their parents, and families. In the intervention and control groups, the mean ± SD infant age at enrollment was 4.76 ± 1.37 d versus 4.69 ± 1.39 d, and the mean LAZ was −1.24 ± 1.07 versus −1.27 ± 1.09, respectively. The maternal duration of schooling [median years (IQR): 8 (0, 10) vs. 8 (2, 10)] and BMI (mean ± SD: 22.70 ± 3.63 vs. 22.56 ± 3.56) were similar for both groups. **Table 3** shows the data on compliance with the maternal snacks and micronutrient supplements among the intervention-group mothers. The mean ± SD days that 1 full packet of snacks was consumed over the 6-mo intervention period was 155.41 ± 35.19, and the proportion of mothers who consumed packets on >75% of the days was 83.8%. For MMNs, the mean ± SD number of days that 1 tablet was consumed was 146.54 ± 34.53 and the proportion of mothers who consumed a tablet for >75% of the days was 78.7%.

**TABLE 2:** Baseline characteristics of enrolled infants and their families, by study group^1^

Variables	Intervention (*n* = 408)	Control (*n* = 408)
Infant characteristics at enrollment (age 0–7 d)		
Age in days at enrollment	4.76 ± 1.37	4.69 ± 1.39
Birth weight, reported or documented,^2^ kg	2.77 ± 0.42	2.74 ± 0.44
Gestational age,^3^ wk	38.8 ± 1.66	38.7 ± 1.81
Prematurity, *n* (%)	30 (7.4)	48 (11.8)
Low birth weight as <2500 g at enrollment, *n* (%)	114 (28.0)	111 (27.2)
Delivered at home, *n* (%)	87 (21.3)	70 (17.2)
Number of male infants, *n* (%)	207 (50.7)	217 (53.2)
Breastfeeding initiated <1 h of birth, *n* (%)	117 (28.7)	115 (28.2)
Age at initiation of breastfeeding, median (IQR), h	2 (0, 6)	2 (0, 5)
Weight,^4^ kg	2.74 ± 0.43	2.74 ± 0.44
Length,^4^ cm	48.03 ± 2.07	47.94 ± 2.25
LAZ using WHO Growth Standards	−1.24 ± 1.07	−1.27 ± 1.09
Stunting as <2 LAZ, *n* (%)	93 (22.9)	96 (23.5)
WLZ score using WHO Growth Standards^5^	−1.00 ± 1.03	−0.97 ± 1.06
Wasting as <2 WLZ,^5^*n* (%)	59 (15.7)	51 (13.8)
WAZ score using WHO Growth Standards	−1.40 ± 1.03	−1.40 ± 1.02
Underweight as <2 WAZ, *n* (%)	102 (25.1)	104 (25.5)
BMI-Z using WHO Growth Standards	−1.24 ± 1.02	−1.20 ± 1.03
Midupper arm circumference, cm	9.43 ± 0.84	9.41 ± 0.81
Head circumference, cm	33.16 ± 1.31	33.15 ± 1.54
Sociodemographic characteristics		
Wealth quintile, *n* (%)		
Poorest	87 (21.3)	77 (18.8)
Very poor	84 (20.6)	79 (19.4)
Poor	88 (21.6)	75 (18.4)
Less poor	75 (18.4)	88 (21.6)
Least poor	74 (18.1)	89 (21.8)
Annual family income, median (IQR), US$	1580 (1580, 3160)	1580 (1422, 3160)
Nuclear family, *n* (%)	189 (46.3)	167 (40.9)
Religion		
Hindu, *n* (%)	324 (79.4)	326 (79.9)
Maternal characteristics		
Age, y	24.43 ± 3.74	24.54 ± 3.72
Duration of schooling, median (IQR), y	8 (0, 10)	8 (2, 10)
Never been to school, *n* (%)	118 (28.9)	101 (24.8)
Homemakers, *n* (%)	406 (99.5)	404 (99.0)
BMI, kg/m^2^	22.70 ± 3.63	22.56 ± 3.56
BMI <18.5 kg/m^2^, *n* (%)	34 (8.4)	43 (10.5)
BMI ≥25.0 kg/m^2^, *n* (%)	99 (24.3)	95 (23.3)
MUAC, cm	24.4 ± 2.8	24.2 ± 2.8
Height, cm	151.25 ± 5.36	151.38 ± 5.99
Height <150 cm, *n* (%)	158 (38.7)	162 (39.7)
Paternal characteristics		
Age, y	28.02 ± 4.32	28.21 ± 4.33
Duration of schooling, median (IQR), y	9 (5, 10.5)	8 (5, 12)
Unemployed, *n* (%)	21 (5.2)	23 (5.6)

1 Data are reported as means ± SDs unless stated otherwise. BMI-Z, BMI *z* score; LAZ, length-for-age *z* score; MUAC, midupper arm circumference; WAZ, weight-for-age *z* score; WLZ, weight-for-length *z* score.

2 Data available for 319 infants in the intervention group and 336 infants in the control group.

3 Documented gestational age (ultrasound/antenatal card/reported months of gestation at birth).

4 One mother–infant dyad in the intervention group refused anthropometric measurements after providing consent.

5 WLZ scores could not be calculated for 32 and 38 infants in the intervention and control groups, respectively, as length at enrollment was <45 cm. The WHO anthropometric *z*-score calculator has no provision to estimate WLZ for length <45 cm.

**TABLE 3:** Compliance with maternal snacks and multiple micronutrients^1^

	Values
One full supplement packet consumed by the mother in days, mean ± SD	155.4 ± 35.1
Number (%) of days where a full-packet supplement was consumed	
>75%	342 (83.8)
51–75%	47 (11.5)
26–50%	12 (2.9)
≤25%	7 (1.7)
Daily micronutrient tablet consumed by the mother in days, mean ± SD	146.5 ± 34.5
Number (%) of days where 1 tablet of the micronutrient was consumed	
>75%	321 (78.6)
51–75%	64 (15.6)
26–50%	8 (1.9)
≤25%	15 (3.6)

1 *n* = 408 enrolled mother–infant dyads.

The mean ± SD LAZ at 6 mo in the intervention and control groups was −0.89 ± 1.12 and −1.0 ± 1.01, respectively, with no significant difference in effect on LAZ (adjusted mean difference: 0.09; 95% CI: −0.03, 0.20). We noted no significant effect of the intervention on other anthropometric outcomes at 6 mo of age. The changes in LAZ between enrollment to 3 mo (adjusted mean difference: 0.03; 95% CI: −0.07, 0.12) and 3 to 6 mo of age (adjusted mean difference: 0.06; 95% CI: −0.04, 0.16) were also similar (**Table 4**). Maternally reported exclusive breastfeeding was higher in the intervention group relative to the control group at 5 mo (45.1% vs. 34.5%; RR: 1.31; 95% CI: 1.04, 1.64; *P* = 0.02) (**Table 5**). There were no significant effects of the intervention on mean hemoglobin concentration or proportion with anemia among infants in the 2 study groups.

**TABLE 4:** Effect of maternal nutritional supplementation during lactation on infant growth^1^

	Measure		Unadjusted risk ratio or unadjusted mean difference (95% CI)	Adjusted risk ratio or adjusted mean difference (95% CI)^2^
	Intervention (*n* = 408)	Control (*n* = 408)		
Attained anthropometric measures at 6 mo of infant age^3^				
Primary outcome				
LAZ at 6 mo (*n* = 388, 384)	−0.89 ± 1.12	−1.0 ± 1.01	0.10 (−0.05, 0.25)	0.09 (−0.03, 0.20)
Secondary outcomes				
WAZ at 6 mo (*n* = 388, 384)	−1.08 ± 1.16	−1.17 ± 1.07	0.08 (−0.08, 0.24)	0.08 (−0.05, 0.21)
WLZ at 6 mo (*n* = 388, 384)	−0.63 ± 1.16	−0.66 ± 1.06	0.03 (−0.12, 0.19)	0.04 (−0.11, 0.20)
Showed stunting at 6 mo (*n* = 388, 384)	59 (15.2)	58 (15.1)	1.00 (0.70,1.45)	1.09 (0.75,1.59)
Showed wasting at 6 mo (*n* = 388, 384)	47 (12.1)	36 (9.4)	1.29 (0.84, 1.99)	1.29 (0.83, 1.99)
Underweight at 6 mo (*n* = 388, 384)	73 (18.8)	77 (20.1)	0.94 (0.68, 1.29)	0.96 (0.70, 1.33)
MUAC *z* score at 6 mo (*n* = 388, 384)	−0.53 ± 1.06	−0.56 ± 0.99	0.03 (−0.12, 0.17)	0.03 (−0.11, 0.16)
Head circumference *z* score at 6 mo in cm, (*n* = 388, 384)	−1.39 ± 1.01	−1.45 ± 1.06	0.06 (−0.09, 0.20)	0.04 (−0.09, 0.17)
Change in anthropometric measures over the 6-mo period^4^				
Change in LAZ at 0–3 mo (*n* = 386, 367)	0.16 ± 0.72	0.15 ± 0.74	0.01 (−0.09, 0.11)	0.03 (−0.07, 0.12)
Change in LAZ at 3–6 mo (*n* = 378, 361)	0.16 ± 0.66	0.11 ± 0.66	0.04 (−0.06, 0.14)	0.06 (−0.04, 0.16)
Mean 3-monthly change in LAZ			0.03 (−0.04, 0.09)	0.04 (−0.02, 0.10)
Change in WLZ at 0–3 mo (*n* = 358, 334)	0.48 ± 1.21	0.45 ± 1.38	0.03 (−0.13, 0.19)	0.04 (−0.10, 0.19)
Change in WLZ at 3–6 mo (*n* = 378, 361)	−0.18 ± 0.82	−0.14 ± 0.89	−0.03 (−0.19, 0.13)	−0.05 (−0.19, 0.10)
Mean 3-monthly change in WLZ			0.00 (−0.10, 0.10)	0.00 (−0.08, 0.08)
Change in MUAC at 0–3 mo (*n* = 386, 367)	3.29 ± 1.07	3.24 ± 1.09	0.05 (−0.08,0.18)	0.06 (−0.07,0.19)
Change in MUAC at 3–6 mo (*n* = 378, 361)	0.76 ± 0.74	0.80 ± 0.74	−0.05 (−0.18, 0.08)	−0.04 (−0.17, 0.09)
Mean 3-monthly change in MUAC			0.00 (−0.08,0.08)	0.01 (−0.07, 0.09)

1 Data are *n* (%) or mean ± SD, with outcome measures of adjusted risk ratios for the numbers of infants showing stunting, wasting, or underweight, and adjusted mean differences for other growth parameters. None of the *P* values (2-sided) were significant. BMI-Z, BMI *z* score; GEE, generalized estimating equation; GLM, generalized linear model; MUAC, midupper arm circumference; LAZ, length-for-age *z* score; WAZ, weight-for-age *z* score; WLZ, weight-for-length *z* score.

2 Adjusted for wealth quintile, gestational age, and LAZ and BMI-Z at baseline.

3 Analysis using GLM.

4 Analysis using GEE with Gaussian family, identity link, exchangeable correlation; adjusted for wealth quintile, BMI-Z at baseline, and gestational age for LAZ changes; adjusted for wealth quintile, LAZ scores, BMI-Z at baseline and gestational age for WLZ changes; adjusted for wealth quintile, LAZ, and BMI-Z at baseline and gestational age for MUAC changes.

**TABLE 5:** Effect of maternal nutritional supplementation during lactation on breastfeeding practices, maternal BMI, and biochemical outcomes^1^

	Measure		Adjusted risk ratio or adjusted mean difference (95% CI)^2^
	Intervention (*n* = 408)	Control (*n* = 408)	
Breastfeeding practices			
Exclusively breastfed (maternally reported)			
At 1 mo (*n* = 390, 392)	325 (83.3)	319 (81.4)	1.02 (0.88, 1.19)
At 3 mo (*n* = 392, 383)	274 (69.9)	244 (63.7)	1.10 (0.92, 1.30)
At 5 mo (*n* = 388, 374)	175 (45.1)	129 (34.5)	1.31 (1.04, 1.64)^3^
Maternal anthropometry at 6 mo			
BMI in kg/m^2^ (*n* = 386, 381)	22.71 ± 3.92	22.13 ± 4.03	0.37 (0.09, 0.64)^3^
MUAC in cm (*n* = 386, 381)	24.98 ± 2.89	24.47 ± 3.0	0.36 (0.12, 0.60)^3^
BMI <18.5 (*n* = 386, 381)	45 (11.7)	63 (16.5)	0.76 (0.50, 1.15)
BMI ≥25 (*n* = 386, 381)	100 (25.9)	91 (23.9)	0.96 (0.71, 1.28)
Mothers’ Hb concentration and proportion with anemia at 6 mo			
Hb in g/dL (*n* = 371, 351)	11.99 ± 1.16	11.62 ± 1.38	0.37 (0.19, 0.56)^3^
Proportion anemic with Hb <12 g/dL (*n* = 371, 351)	147 (39.6)	195 (55.6)	0.71 (0.58, 0.88)^3^
Infants’ Hb concentration and proportion with anemia at 6 mo			
Hb in g/dL (*n* = 368, 351)	10.50 ± 1.22	10.37 ± 1.15	0.13 (−0.05, 0.30)
Proportion anemic with Hb <11 g/dL (*n* = 368, 351)	242 (65.8)	242 (68.9)	0.95 (0.80, 1.14)

1 Data are *n* (%) or mean ± SD, with outcome measures of adjusted risk ratio for proportion with breastfeeding outcomes, maternal BMI <18.5 kg/m^2^, and proportion of infants and mother with anemia; adjusted mean differences for maternal BMI, maternal MUAC, and hemoglobin concentration in infants and mothers. Hb, hemoglobin; MUAC, midupper arm circumference.

2 For breastfeeding proportion/hemoglobin concentration/proportion with anemia of both mother and infants, no adjustments were made; maternal BMI was adjusted for wasting at enrollment, maternal BMI at enrollment, and maternal MUAC at baseline; maternal MUAC was adjusted for maternal BMI at enrollment and maternal MUAC at baseline.

3 Denotes significant 2-sided *P* value (<0.05).

There were some effects of the intervention on the nutritional status of the mothers. In the intervention group, the mean ± SD maternal BMI remained the same during the period of supplementation—that is, 22.70 ± 3.63 at baseline and 22.71 ± 3.92 at an infant age of 6 mo. However, we noted a decline in BMI in the control-group mothers from 22.56 ± 3.56 at baseline to 22.13 ± 4.03 at 6 mo of infant age. The adjusted mean difference in BMI between the 2 groups at the end of 6 mo of supplementation was 0.37 (95% CI: 0.09, 0.64; *P* = 0.009). The mean ± SD maternal MUAC in the intervention and control groups was 24.98 ± 2.89 cm and 24.47 ± 3.0 cm, respectively, with an adjusted mean difference of 0.36 cm (95% CI: 0.12, 0.60 cm; *P* = 0.003) (Table 5). Mothers in the intervention group had a higher hemoglobin concentration (mean ± SD: 11.99 ± 1.16 vs. 11.62 ± 1.38 g/dL) than the control group, with an adjusted mean difference of 0.37 g/dL (95% CI: 0.19, 0.56 g/dL; *P* < 0.001). The anemia (hemoglobin <12 g/dL) rates in the intervention-group mothers were lower than in the control group at 6 mo of infant age (39.6% vs. 55.6%; RR: 0.71; 95% CI: 0.58, 0.88; *P* = 0.002) (Table 5).

In a post hoc exploratory analysis, there was no evidence of a significant effect of the intervention on LAZ at 6 mo of age within the maternal height and BMI categories, or within subgroups based on infant weight at enrollment, or on stunting, wasting, or underweight status at birth.

**Table 6** depicts the findings of the maternal 24-h dietary recalls in both the intervention and control groups at 3 mo of infant age. The mean ± SD intake of calories in kilocalories (2247 ± 775 vs. 2088 ± 874), carbohydrates in grams (304 ± 115 vs. 284 ± 130), proteins in grams (70 ± 26 vs. 64 ± 27), and fats in grams (72 ± 37 vs. 69 ± 41) was slightly higher in the intervention-group mothers compared with the control group; all differences except for fats were statistically significant.

**TABLE 6:** Twenty-four-hour dietary recalls of mothers enrolled in the study at 3 mo of infant age^1^

	Intervention^2^	Control
Number of mothers for whom dietary recalls conducted	395	393
Total calories in kilocalories consumed^3^	2247 ± 775	2088 ± 874
Total carbohydrate in grams consumed^3^	304 ± 115	284 ± 130
Total protein in grams consumed^3^	70 ± 26	64 ± 27
Total fat in grams consumed	72 ± 37	69 ± 41

1 Data are presented as mean ± SD.

2 The supplement is included in the totals for the intervention group.

3 Statistically significant at 2-sided *P* < 0.05.

## Discussion

In this study, we found that maternal supplementation in the first 6 mo postpartum led to a small, nonsignificant improvement in infant linear growth at 6 mo of age. However, supplementation resulted in improvements in maternal health outcomes: BMI, MUAC, hemoglobin concentration, and a lower rate of anemia.

Investigators have over the last 3 decades examined the effect of nutritional supplementation of lactating mothers with respect to breast-milk volume, composition, and infant growth, but these studies have not been conclusive (25–28). Previous studies have shown an effect of maternal supplementation during pregnancy and in the first 6 mo of postnatal life on infant growth outcomes (29–32). The findings from these studies suggested an improvement in birth size/length but did not show a large improvement in infant linear growth or other anthropometric outcomes at 6 mo of age. In a recent network meta-analysis, it was shown that in comparison to standard-of-care, fortified lipid-based nutrient supplementation during pregnancy and the first 6 mo of postnatal life resulted in a mean difference of 0.08 (95% CI: −0.12, 0.29) in LAZ at 6 mo (33). This pooled estimate is similar to the findings of the current study in which we focused only on maternal supplementation during the first 6 mo of lactation.

Evidence from the extant literature suggests an improvement in birth length due to maternal supplementation during pregnancy; however, no such effect of continued maternal supplementation in the lactation period on infant growth in the first 6 mo of life has been noted. We posit the following potential explanations for this. First, the supplementation may not alter breast-milk composition significantly. Although maternal supplementation can increase breast-milk thiamin, riboflavin, iodine, selenium, and vitamin B-6, A, D, E, and K concentrations, most of these are type I nutrients. Other key growth-limiting micronutrients, such as zinc, phosphorus, and magnesium, in breast milk are stable or refractory to maternal intake or supplementation (15, 34, 35). Type I nutrients are those that are required to maintain normal bodily functions and consequently their deficiencies lead to characteristic clinical symptoms associated with dysfunction of a particular biochemical pathway. On the other hand, type II nutrients are those that are required for optimal growth of lean tissues (36, 37).

The available literature also suggests that the protein concentrations are stable from 2 to 6 mo of infant age and unaffected by maternal nutritional status (15). Similarly, the lactose concentrations are fairly consistent, with a small CV (2–4%), and they are independent of maternal diet and nutritional status (15). Fat concentrations also remain stable in mature milk. However, the fatty acid composition—especially of the PUFAs (e.g., DHA)—depends upon the nutritional intake and status of the mother (15). The concentrations of PUFAs in breast milk have also been shown to be positively associated with infant growth (both weight and length) during the first 6 mo of life (38). Second, there may be a need for additional interventions that focus on the prevention of infections and the promotion of optimal infant health care. Third, the lack of any observed effects of supplementation on linear growth may be due to gut infection leading to both local and systemic inflammation, as this study was performed in a setting with poor environmental hygiene, food, and water quality (39, 40). Furthermore, ∼40% of the mothers were of short height (<150 cm), and therefore null effects of supplementation due to intergenerational adversity cannot be ruled out.

Lactation leading to increased nutrient demands and modest improvements in maternal BMI, MUAC, and hemoglobin concentrations in mothers in the intervention group call for focused nutritional programs for lactating mothers. A higher proportion of exclusive breastfeeding due to maternal supplementation has also been shown in previous randomized controlled trials (41, 42). One of the common reasons for introducing food other than breast milk to low-middle-income settings is the perception of inadequate maternal milk production (43–45). It is thus possible that maternal supplementation leads to increased confidence in the mother regarding her milk production. This finding is of particular importance in India—where breastfeeding practices remain suboptimal—and from a public health perspective, it underscores the relevance of coupling nutritional programs for lactating mothers with programs aimed at the promotion of exclusive breastfeeding. An important issue to consider is that infants who are predominantly or exclusively breastfed have a different metabolic profile and micronutrient status relative to those who are either not breastfed or breastfed less frequently. Also, morbidity could be reduced in infants with greater exclusive breastfeeding. In our study, the possibility that the increase in the proportion of infants who were exclusively breastfed in mothers who received nutrient supplementation might influence infant growth outcomes cannot therefore be ruled out.

We did not in the present study find an effect of maternal supplementation on infant hemoglobin status or the proportion of infants with anemia after 6 mo of supplementation. A recent review suggested that breast-milk concentrations of micronutrients and minerals related to iron metabolism and erythropoiesis (such as iron, folate, copper, and zinc) were unaltered despite maternal supplementation (15). Additionally, concentrations of thiamin and vitamin B-12 were increased in breast milk upon maternal supplementation only in the case of maternal dietary insufficiency (15). These findings could possibly explain the lack of an observed effect on infant hemoglobin and anemia status despite an improvement in maternal hemoglobin and reduction in anemia status.

The strengths of our study included a rigorous study design and low loss-to-follow-up rates. Outcomes were assessed by a trained and standardized team. Although it was difficult to ensure complete blinding due to the nature of the intervention, there was minimal contact between supplement delivery and independent outcome-ascertainment teams. Our study entailed a few limitations. First, while bias due to lack of blinding was, to some extent, alleviated by having different teams measuring the outcomes and delivering the interventions, this did not remove any bias introduced before the outcomes were measured. The possibility of behavioral modification(s)—such as those related to exclusive breastfeeding—could therefore not be discounted. Such behavioral modifications might have potentially introduced other actions that could have influenced child growth in either direction. In addition, the proportion of infants who were exclusively breastfed might have been overreported, as these were ascertained through maternal recall. Second, while direct observation of the consumption of snacks and MMNs would have been ideal, the assessment of compliance with respect to snacks and MMNs was reported. Third, this study had limited power to detect a small effect size. For the 0.10-SD difference in the mean LAZ that we found in our study, the current sample size provided ∼40% power for detecting a difference. Finally, the 24-h dietary recalls indicated some displacement of the regular meals; however, compliance with the supplement was high—84% of the mothers consumed a full-supplement packet for >75% of days.

In conclusion, the present study does not support our hypothesis that additional supplementation of energy, protein, and micronutrients during lactation has a modest impact on the growth of infants during their first 6 mo of life. We, however, noted a trend towards improved linear growth that failed to reach statistical significance. The findings support the provision of nutritional supplementation to lactating mothers in order to improve infant growth.

## Supplementary Material

nqaa383_Supplement_File

## Data Availability

The data described in the manuscript, code book, and analytic code will not be made available. The organization conducting the trial (Society for Applied Studies, India) is a collaborator in the Healthy Birth, Growth, and Development Knowledge Integration (HBGDKi) initiative launched by the Bill & Melinda Gates Foundation, and the data generated from the study will be shared as part of the HBGDKi repository (https://github.com/HBGDki). However, individual requests can be considered on a case-by-case basis. The request for data should be accompanied by a detailed proposal describing the intended scientific question(s) to be addressed. Proposals should be submitted to Dr. Sunita Taneja (sunita.taneja@sas.org.in).

## Notes

The present study received funding from the Bill & Melinda Gates Foundation through the Biotechnology Industry Research Assistance Council (BIRAC) of the Department of Biotechnology, Government of India (OPP1177843). We acknowledge the support of the Vitamin Angel Alliance, Inc. (Vitamin Angels), California, for donating the micronutrient supplements for use in the study. The funders had no role in the study design, data collection or analysis, decision to publish, or preparation of the manuscript.

Supplemental Table 1 is available from the “Supplementary data” link in the online posting of the article and from the same link in the online table of contents at https://academic.oup.com/ajcn/.

Abbreviations used: GEE, generalized estimating equation; GLM, generalized linear model; LAZ, length-for-age *z* score; MMN, multiple micronutrient; MUAC, midupper arm circumference; MUAC-Z, midupper arm circumference *z* score; TAG, Technical Advisory Group; WAZ, weight-for-age *z* score; WLZ, weight-for-length *z* score.
