# Supplementary material for: Impact of nutritional interventions among lactating mothers on the growth of their infants in the first 6 months of life: a randomized controlled trial in Delhi, India
Source: Am J Clin Nutr. 2021 Feb 10;113(4):884–94. doi: 10.1093/ajcn/nqaa383 (PMC8023824; doi:10.1093/ajcn/nqaa383)
Supplement: nqaa383_Supplement_File [file nqaa383_supplement_file.pdf]

Impact of nutritional interventions among lactating mothers on the growth of their infants in the first 6 months of life: a randomized controlled trial in Delhi, India

Sunita Taneja

ONLINE SUPPLEMENTARY MATERIAL

SUPPLEMENTARY TABLE 1 Composition of the multiple micronutrient formulation

**Nutrients**	**Composition of Vitamin Angels tablets**	**%RDA WHO***
Vitamin A (μg/d)	800	94
Vitamin D (μg/d)	5	100
Vitamin K (μg/d)	-	
Vitamin C (μg/d)	70	100
Vitamin E (μg a-TE/d)	10	133
Thiamine (mg/d)	1.4	93
Riboflavin (mg/d)	1.4	88
Niacin (mg/d)	18	106
Pyridoxine (mg/d)	1.9	95
Pantothenate (mg/d)	-	
Folate (μg/d)	400	80
Vitamin B12 (μg/d)	2.6	93
Biotin (μg/d)	-	
Iron (mg/d)	30	200
Calcium (mg/d)	-	
Zinc (mg/d)	15	158
Iodine (μg/d)	150	75
Selenium (μg/d)	65	186

*UNICEF/UNU/WHO. Composition of a multi-micronutrient supplement to be used in pilot programs among pregnant women in developing countries. New York, NY: UNICEF, 1999
